# Supplementary material for: End Sequence Analysis Toolkit (ESAT) expands the extractable information from single-cell RNA-seq data
Source: Genome Res. 2016 Oct;26(10):1397–410. doi: 10.1101/gr.207902.116 (PMC5052061; doi:10.1101/gr.207902.116)
Supplement: Supplemental Material [file supp_gr.207902.116_Supp_Materials.tar.gz › ESAT-esat_v0.1/doc/html/classumms_1_1core_1_1alignment_1_1_single_end_alignment.html]

ESAT: umms.core.alignment.SingleEndAlignment Class Reference


ESAT


- Main Page
- Related Pages
- Packages
- Classes
- Files

- Class List
- Class Index
- Class Hierarchy
- Class Members

All Classes Namespaces Files Functions Variables Enumerator Pages

- umms
- core
- alignment
- SingleEndAlignment

Classes |
Public Member Functions |
List of all members

umms.core.alignment.SingleEndAlignment Class Reference

Inheritance diagram for umms.core.alignment.SingleEndAlignment:

[legend]

Collaboration diagram for umms.core.alignment.SingleEndAlignment:

[legend]

|  |  |
| --- | --- |
| Classes | |
| class | WrapSamRecord |
|  | |

|  |  |
| --- | --- |
| Public Member Functions | |
|  | SingleEndAlignment (SAMRecord read) |
|  | |
|  | SingleEndAlignment (org.broad.igv.sam.Alignment read) |
|  | |
|  | SingleEndAlignment (SAMRecord read, boolean isFirstMateFlag) |
|  | |
|  | SingleEndAlignment () |
|  | |
| void | setSplicedEdges (Collection< Annotation > se) |
|  | |
| void | setHasIndel (boolean h) |
|  | |
| String | toString () |
|  | |
| void | setIsFirstMate (boolean value) |
|  | |
| boolean | getIsFirstMate () |
|  | |
| int | getReadLength () |
|  | |
| String | getReadName () |
|  | |
| String | getChromosome () |
|  | |
| int | getFragmentStart () |
|  | |
| int | getMappingQuality () |
|  | |
| boolean | isPaired () |
|  | |
| boolean | isChimera () |
|  | |
| int | getFragmentEnd () |
|  | |
| Collection< Integer > | getFragmentSize (CoordinateSpace C) |
|  | |
| Collection<?extends Window > | getFragment (CoordinateSpace C) |
|  | |
| Strand | getFragmentStrand () |
|  | |
| int | getLastFragmentPositionStranded () |
|  | |
| int | getFirstFragmentPositionStranded () |
|  | |
| boolean | isDuplicate () |
|  | |
| void | setDuplicateFlag (boolean duplicateFlag) |
|  | |
| Annotation | getReadAlignmentBlocks (CoordinateSpace C) |
|  | |
| String | getChr () |
|  | |
| int | getAlignmentStart () |
|  | |
| int | getAlignmentEnd () |
|  | |
| boolean | isMapped () |
|  | |
| Object | getAttribute (String string) |
|  | |
| String | getReadSequence () |
|  | |
| double | getWeight () |
|  | |
| boolean | isProperPair () |
|  | |
| void | setProperPairFlag (boolean properPairFlag) |
|  | |
| final SAMRecord | toSAMRecord () |
|  | |
| String | getCigarString () |
|  | |
| Collection< Annotation > | getReadAlignments (CoordinateSpace space) |
|  | |
| Collection<?extends Annotation > | getSpliceConnections () |
|  | |
| boolean | equals (Annotation other) |
|  | |
| Collection< Alignment > | getReadMates () |
|  | |
| void | setFragmentStrand (TranscriptionRead strand) |
|  | |
| boolean | hasIndel () |
|  | |
| int[] | getIntervalBetweenReads () |
|  | |
| int | getFragmentMidpoint (Annotation annot) |
|  | |
| void | setHeader (SAMFileHeader header) |
|  | |
| SAMFileHeader | getHeader () |
|  | |
| Public Member Functions inherited from umms.core.annotation.BasicAnnotation | |
|  | BasicAnnotation () |
|  | |
|  | BasicAnnotation (SAMRecord samRecord) |
|  | |
|  | BasicAnnotation (String ucsc) |
|  | |
|  | BasicAnnotation (String referenceName, int start, int end, Strand orientation, String name) |
|  | |
|  | BasicAnnotation (String referenceName, Strand orientation, String name, Collection<?extends Annotation > blocks) |
|  | |
|  | BasicAnnotation (String referenceName, int start, int end) |
|  | |
|  | BasicAnnotation (String referenceName, int start, int end, Strand orientation) |
|  | |
|  | BasicAnnotation (String referenceName, int start, int end, String orientation) |
|  | |
|  | BasicAnnotation (BasicAnnotation other) |
|  | |
|  | BasicAnnotation (Annotation other) |
|  | |
|  | BasicAnnotation (Collection<?extends Annotation > blocks) |
|  | |
|  | BasicAnnotation (Collection<?extends Annotation > blocks, Strand orientation, String name) |
|  | |
|  | BasicAnnotation (Collection<?extends Annotation > blocks, String name) |
|  | |
|  | BasicAnnotation (String referenceName, CompoundInterval blocks, Strand orientation, String name) |
|  | |
|  | BasicAnnotation (String referenceName, CompoundInterval blocks, Strand orientation) |
|  | |
|  | BasicAnnotation (String referenceName, CompoundInterval blocks) |
|  | |
| Annotation | copy () |
|  | |
| int | getStart () |
|  | |
| int | getEnd () |
|  | |
| int | getMidpoint () |
|  | |
| String | getReferenceName () |
|  | |
| String | getName () |
|  | |
| Strand | getOrientation () |
|  | |
| boolean | isUnoriented () |
|  | |
| List<?extends Annotation > | getBlocks () |
|  | |
| List<?extends Annotation > | getBlocks (boolean oriented) |
|  | |
| Annotation[] | getFlankingBlocks (Annotation spliceJunction) |
|  | |
| int | numBlocks () |
|  | |
| int | length () |
|  | |
| int | getReferenceCoordinateAtPosition (int positionInAnnotation, boolean ignoreOrientation) |
|  | |
| int | getPositionAtReferenceCoordinate (int referenceCoordinate, boolean ignoreOrientation) |
|  | |
| double | getScore () |
|  | |
| String | toString () |
|  | |
| void | setStart (int start) |
|  | |
| void | setEnd (int end) |
|  | |
| void | setOrientation (Strand orientation) |
|  | |
| void | setOrientedStart (int orientedStart) |
|  | |
| void | setOrientedEnd (int orientedEnd) |
|  | |
| void | setReferenceName (String refName) |
|  | |
| void | setName (String n) |
|  | |
| void | setScore (double s) |
|  | |
| void | addBlocks (Annotation block) |
|  | |
| void | addBlocks (Collection<?extends Annotation > blocks) |
|  | |
| void | shift (int delta) |
|  | |
| void | moveToCoordinate (int coordinateInReference) |
|  | |
| boolean | overlaps (Annotation other, int buffer, boolean considerOrientation) |
|  | |
| boolean | overlaps (List<?extends Annotation > otherBlocks, int buffer) |
|  | |
| int | getOverlap (Annotation other) |
|  | |
| boolean | contains (Annotation other) |
|  | |
| Annotation | union (Annotation other) |
|  | |
| Annotation | intersect (Annotation other) |
|  | |
| List< Annotation > | disect (Annotation a) |
|  | |
| List< Annotation > | disect (List<?extends Annotation > disectors) |
|  | |
| Annotation | minus (Annotation other) |
|  | |
| Annotation | minus (Collection<?extends Annotation > others) |
|  | |
| void | stitchTo (Annotation next) |
|  | |
| Annotation | complement () |
|  | |
| Collection<?extends Annotation > | getSpliceConnections () |
|  | |
| Public Member Functions inherited from umms.core.annotation.AbstractAnnotation | |
| int | size () |
|  | |
| int | getSize () |
|  | |
| int | getMidpoint () |
|  | |
| Strand | getStrand () |
|  | |
| boolean | hasOrientation () |
|  | |
| boolean | isNegativeStrand () |
|  | |
| String | getChr () |
|  | |
| int | getOrientedStart () |
|  | |
| int | getOrientedEnd () |
|  | |
| int | getLengthOnReference () |
|  | |
| int | getReferenceCoordinateAtPosition (int positionInAnnotation) |
|  | |
| int | getPositionAtReferenceCoordinate (int referenceCoordinate) |
|  | |
| void | setOrientation (char orientation) |
|  | |
| void | expand (int deltaStart, int deltaEnd) |
|  | |
| Annotation | trim (int deltaStart, int deltaEnd) |
|  | |
| boolean | fullyContains (Annotation other) |
|  | |
| boolean | overlaps (Annotation other) |
|  | |
| boolean | overlaps (Annotation other, int buffer) |
|  | |
| boolean | overlapsStranded (Annotation other) |
|  | |
| boolean | overlaps (Annotation other, boolean considerOrientation) |
|  | |
| boolean | overlaps (Collection<?extends Annotation > others) |
|  | |
| boolean | overlaps (Collection<?extends Annotation > others, int buffer) |
|  | |
| String | toUCSC () |
|  | |
| String | toBED () |
|  | |
| final String | getFullInfoString () |
|  | |
| String | toBED (int r, int g, int b) |
|  | |
| String | toShortBED () |
|  | |
| String | toBEDGraph () |
|  | |
| int | getDistanceTo (Annotation other) |
|  | |
| String | toCigar () |
|  | |
| List< Annotation > | intersect (List<?extends Annotation > others) |
|  | |
| int | compareTo (Annotation other) |
|  | |
| int | compareToAnnotation (Annotation b) |
|  | |
| int | compareToAnnotation (Annotation b, boolean useOrientation) |
|  | |
| boolean | equals (Annotation a) |
|  | |
| boolean | equals (Annotation a, boolean useOrientation) |
|  | |
| int | getSAMStart () |
|  | |
| int | getSAMEnd () |
|  | |
| Public Member Functions inherited from umms.core.alignment.Alignment | |
| boolean | isNegativeStrand () |
|  | |
| int | getStart () |
|  | |
| int | getEnd () |
|  | |

|  |  |
| --- | --- |
| Additional Inherited Members | |
| Static Public Member Functions inherited from umms.core.annotation.BasicAnnotation | |
| static Annotation | createFromUCSC (String ucsc) |
|  | |
| static BasicAnnotation | fromFullInfoString (String fullInfoString) |
|  | |
| static void | main (String[] args) throws IOException |
|  | |
| Static Public Member Functions inherited from umms.core.annotation.AbstractAnnotation | |
| static Strand | getStrand (String orientation) |
|  | |
| static Strand | getStrand (char orientation) |
|  | |
| Static Public Attributes inherited from umms.core.annotation.AbstractAnnotation | |
| static final int | MAX\_DISTANCE = 1000000000 |
|  | |
| Protected Attributes inherited from umms.core.annotation.BasicAnnotation | |
| CompoundInterval | blocks = new CompoundInterval() |
|  | |

## Constructor & Destructor Documentation

|  |  |  |  |  |  |
| --- | --- | --- | --- | --- | --- |
| umms.core.alignment.SingleEndAlignment.SingleEndAlignment | ( | SAMRecord | *read* | ) |  |

Here is the call graph for this function:

|  |  |  |  |  |  |
| --- | --- | --- | --- | --- | --- |
| umms.core.alignment.SingleEndAlignment.SingleEndAlignment | ( | org.broad.igv.sam.Alignment | *read* | ) |  |

This is to populate our Alignment from the old legacy IGV alignments

Parameters
:   |  |  |
    | --- | --- |
    | read |  |

Here is the call graph for this function:

|  |  |  |  |
| --- | --- | --- | --- |
| umms.core.alignment.SingleEndAlignment.SingleEndAlignment | ( | SAMRecord | *read*, |
|  |  | boolean | *isFirstMateFlag* |
|  | ) |  |  |

Here is the call graph for this function:

|  |  |  |  |  |
| --- | --- | --- | --- | --- |
| umms.core.alignment.SingleEndAlignment.SingleEndAlignment | ( |  | ) |  |

## Member Function Documentation

|  |  |  |  |  |  |
| --- | --- | --- | --- | --- | --- |
| boolean umms.core.alignment.SingleEndAlignment.equals | ( | Annotation | *other* | ) |  |

Implements umms.core.annotation.Annotation.

Here is the call graph for this function:

|  |  |  |  |  |
| --- | --- | --- | --- | --- |
| int umms.core.alignment.SingleEndAlignment.getAlignmentEnd | ( |  | ) |  |

Returns the end position of the alignment

Returns

Implements umms.core.alignment.Alignment.

Here is the call graph for this function:

Here is the caller graph for this function:

|  |  |  |  |  |
| --- | --- | --- | --- | --- |
| int umms.core.alignment.SingleEndAlignment.getAlignmentStart | ( |  | ) |  |

Returns the start position of the alignment

Returns

Implements umms.core.alignment.Alignment.

Here is the call graph for this function:

Here is the caller graph for this function:

|  |  |  |  |  |  |
| --- | --- | --- | --- | --- | --- |
| Object umms.core.alignment.SingleEndAlignment.getAttribute | ( | String | *string* | ) |  |

Returns the value for the specified attribute

Parameters
:   |  |  |
    | --- | --- |
    | string |  |

Returns

Implements umms.core.alignment.Alignment.

|  |  |  |  |  |
| --- | --- | --- | --- | --- |
| String umms.core.alignment.SingleEndAlignment.getChr | ( |  | ) |  |

Get chromosome

Returns

Implements umms.core.alignment.Alignment.

Here is the call graph for this function:

Here is the caller graph for this function:

|  |  |  |  |  |
| --- | --- | --- | --- | --- |
| String umms.core.alignment.SingleEndAlignment.getChromosome | ( |  | ) |  |

Returns the chromosome for this alignment

Here is the call graph for this function:

|  |  |  |  |  |
| --- | --- | --- | --- | --- |
| String umms.core.alignment.SingleEndAlignment.getCigarString | ( |  | ) |  |

|  |  |  |  |  |
| --- | --- | --- | --- | --- |
| int umms.core.alignment.SingleEndAlignment.getFirstFragmentPositionStranded | ( |  | ) |  |

Get the beginning position of the read considering strand

Returns
:   Highest position if negative strand, lowest position otherwise

Implements umms.core.alignment.Alignment.

Here is the call graph for this function:

|  |  |  |  |  |  |
| --- | --- | --- | --- | --- | --- |
| Collection<? extends Window> umms.core.alignment.SingleEndAlignment.getFragment | ( | CoordinateSpace | *C* | ) |  |

Returns an object for the fragment from start to end of alignment in the specified coordinate space

Implements umms.core.alignment.Alignment.

Here is the call graph for this function:

|  |  |  |  |  |
| --- | --- | --- | --- | --- |
| int umms.core.alignment.SingleEndAlignment.getFragmentEnd | ( |  | ) |  |

Returns the end of the fragment

Implements umms.core.alignment.Alignment.

Here is the call graph for this function:

Here is the caller graph for this function:

|  |  |  |  |  |  |
| --- | --- | --- | --- | --- | --- |
| int umms.core.alignment.SingleEndAlignment.getFragmentMidpoint | ( | Annotation | *annot* | ) |  |

Get midpoint of fragment with respect to coordinate space

Parameters
:   |  |  |
    | --- | --- |
    | annot | Parent annotation |

Returns
:   Fragment midpoint in coordinate space

Implements umms.core.alignment.Alignment.

|  |  |  |  |  |  |
| --- | --- | --- | --- | --- | --- |
| Collection<Integer> umms.core.alignment.SingleEndAlignment.getFragmentSize | ( | CoordinateSpace | *C* | ) |  |

Returns the size of the fragment

Implements umms.core.alignment.Alignment.

Here is the call graph for this function:

|  |  |  |  |  |
| --- | --- | --- | --- | --- |
| int umms.core.alignment.SingleEndAlignment.getFragmentStart | ( |  | ) |  |

Returns the start of the fragment

Implements umms.core.alignment.Alignment.

Here is the call graph for this function:

Here is the caller graph for this function:

|  |  |  |  |  |
| --- | --- | --- | --- | --- |
| Strand umms.core.alignment.SingleEndAlignment.getFragmentStrand | ( |  | ) |  |

Returns the strand for the fragment, which is same as read strand

Implements umms.core.alignment.Alignment.

Here is the caller graph for this function:

|  |  |  |  |  |
| --- | --- | --- | --- | --- |
| SAMFileHeader umms.core.alignment.SingleEndAlignment.getHeader | ( |  | ) |  |

Returns the SAM file header for this alignment

Returns

Implements umms.core.alignment.Alignment.

|  |  |  |  |  |
| --- | --- | --- | --- | --- |
| int [] umms.core.alignment.SingleEndAlignment.getIntervalBetweenReads | ( |  | ) |  |

Get the coordinates of the first and last position of the interval between the reads

Returns
:   The coordinates of the space between the reads or null if single read or reads overlap

Implements umms.core.alignment.Alignment.

|  |  |  |  |  |
| --- | --- | --- | --- | --- |
| boolean umms.core.alignment.SingleEndAlignment.getIsFirstMate | ( |  | ) |  |

|  |  |  |  |  |
| --- | --- | --- | --- | --- |
| int umms.core.alignment.SingleEndAlignment.getLastFragmentPositionStranded | ( |  | ) |  |

Get the ending position of the read considering strand

Returns
:   Lowest position if negative strand, highest position otherwise

Implements umms.core.alignment.Alignment.

Here is the call graph for this function:

|  |  |  |  |  |
| --- | --- | --- | --- | --- |
| int umms.core.alignment.SingleEndAlignment.getMappingQuality | ( |  | ) |  |

Returns the mapping quality of the alignment

Implements umms.core.alignment.Alignment.

Here is the caller graph for this function:

|  |  |  |  |  |  |
| --- | --- | --- | --- | --- | --- |
| Annotation umms.core.alignment.SingleEndAlignment.getReadAlignmentBlocks | ( | CoordinateSpace | *C* | ) |  |

Returns the alignment Blocks

Implements umms.core.alignment.Alignment.

|  |  |  |  |  |  |
| --- | --- | --- | --- | --- | --- |
| Collection<Annotation> umms.core.alignment.SingleEndAlignment.getReadAlignments | ( | CoordinateSpace | *space* | ) |  |

This will return the actual read alignments

Parameters
:   |  |  |
    | --- | --- |
    | space | Coordinate Space to return the blocks in |

Implements umms.core.alignment.Alignment.

|  |  |  |  |  |
| --- | --- | --- | --- | --- |
| int umms.core.alignment.SingleEndAlignment.getReadLength | ( |  | ) |  |

Returns the length of the read

Returns

Here is the call graph for this function:

|  |  |  |  |  |
| --- | --- | --- | --- | --- |
| Collection<Alignment> umms.core.alignment.SingleEndAlignment.getReadMates | ( |  | ) |  |

Returns the alignment object

Returns

Implements umms.core.alignment.Alignment.

|  |  |  |  |  |
| --- | --- | --- | --- | --- |
| String umms.core.alignment.SingleEndAlignment.getReadName | ( |  | ) |  |

Returns the name of this read pair.

Implements umms.core.alignment.Alignment.

Here is the call graph for this function:

Here is the caller graph for this function:

|  |  |  |  |  |
| --- | --- | --- | --- | --- |
| String umms.core.alignment.SingleEndAlignment.getReadSequence | ( |  | ) |  |

Implements umms.core.alignment.Alignment.

Here is the caller graph for this function:

|  |  |  |  |  |
| --- | --- | --- | --- | --- |
| Collection<? extends Annotation> umms.core.alignment.SingleEndAlignment.getSpliceConnections | ( |  | ) |  |

Return the splice connections contained within this read

Returns

Implements umms.core.alignment.Alignment.

|  |  |  |  |  |
| --- | --- | --- | --- | --- |
| double umms.core.alignment.SingleEndAlignment.getWeight | ( |  | ) |  |

The weight to give this alignment in the scoring. For example, if you want to scale by number of hits, the weight would be 1/NH

Returns

Implements umms.core.alignment.Alignment.

|  |  |  |  |  |
| --- | --- | --- | --- | --- |
| boolean umms.core.alignment.SingleEndAlignment.hasIndel | ( |  | ) |  |

Returns whether the read contains an insertion or deletion

Returns

Implements umms.core.alignment.Alignment.

|  |  |  |  |  |
| --- | --- | --- | --- | --- |
| boolean umms.core.alignment.SingleEndAlignment.isChimera | ( |  | ) |  |

For single end, returns false. For paired end, returns true if reads successfully map to different chromosomes.

Returns

Implements umms.core.alignment.Alignment.

|  |  |  |  |  |
| --- | --- | --- | --- | --- |
| boolean umms.core.alignment.SingleEndAlignment.isDuplicate | ( |  | ) |  |

Returns true is the alignment pair is a duplicate

Implements umms.core.alignment.Alignment.

|  |  |  |  |  |
| --- | --- | --- | --- | --- |
| boolean umms.core.alignment.SingleEndAlignment.isMapped | ( |  | ) |  |

Returns true if the alignment is mapped

Returns

Implements umms.core.alignment.Alignment.

|  |  |  |  |  |
| --- | --- | --- | --- | --- |
| boolean umms.core.alignment.SingleEndAlignment.isPaired | ( |  | ) |  |

Always returns false for objects of this class.

Implements umms.core.alignment.Alignment.

|  |  |  |  |  |
| --- | --- | --- | --- | --- |
| boolean umms.core.alignment.SingleEndAlignment.isProperPair | ( |  | ) |  |

Returns whether the alignment represented a proper pair defined by the aligner

Returns

Implements umms.core.alignment.Alignment.

|  |  |  |  |  |  |
| --- | --- | --- | --- | --- | --- |
| void umms.core.alignment.SingleEndAlignment.setDuplicateFlag | ( | boolean | *duplicateFlag* | ) |  |

Sets the duplicate flag

Implements umms.core.alignment.Alignment.

|  |  |  |  |  |  |
| --- | --- | --- | --- | --- | --- |
| void umms.core.alignment.SingleEndAlignment.setFragmentStrand | ( | TranscriptionRead | *strand* | ) |  |

Sets the strand for the fragment to the strand passed as argument

Parameters
:   |  |  |
    | --- | --- |
    | strand | TODO: CHECK IF THIS IS CORRECT |

Implements umms.core.alignment.Alignment.

|  |  |  |  |  |  |
| --- | --- | --- | --- | --- | --- |
| void umms.core.alignment.SingleEndAlignment.setHasIndel | ( | boolean | *h* | ) |  |

|  |  |  |  |  |  |
| --- | --- | --- | --- | --- | --- |
| void umms.core.alignment.SingleEndAlignment.setHeader | ( | SAMFileHeader | *header* | ) |  |

Sets the SAM File header for this alignment

Parameters
:   |  |  |
    | --- | --- |
    | header |  |

Implements umms.core.alignment.Alignment.

|  |  |  |  |  |  |
| --- | --- | --- | --- | --- | --- |
| void umms.core.alignment.SingleEndAlignment.setIsFirstMate | ( | boolean | *value* | ) |  |

Will set the value of the isFirstMate flag

Parameters
:   |  |  |
    | --- | --- |
    | value | true: this unpaired mate was the first mate false: this unpaired mate was second mate |

Here is the caller graph for this function:

|  |  |  |  |  |  |
| --- | --- | --- | --- | --- | --- |
| void umms.core.alignment.SingleEndAlignment.setProperPairFlag | ( | boolean | *properPairFlag* | ) |  |

Set whether the alignment represented a proper pair defined by the aligner

Returns

Implements umms.core.alignment.Alignment.

|  |  |  |  |  |  |
| --- | --- | --- | --- | --- | --- |
| void umms.core.alignment.SingleEndAlignment.setSplicedEdges | ( | Collection< Annotation > | *se* | ) |  |

|  |  |  |  |  |
| --- | --- | --- | --- | --- |
| final SAMRecord umms.core.alignment.SingleEndAlignment.toSAMRecord | ( |  | ) |  |

Implements umms.core.alignment.Alignment.

Here is the call graph for this function:

|  |  |  |  |  |
| --- | --- | --- | --- | --- |
| String umms.core.alignment.SingleEndAlignment.toString | ( |  | ) |  |

Here is the call graph for this function:

---

The documentation for this class was generated from the following file:

- java/umms/core/alignment/SingleEndAlignment.java


---

Generated on Fri Aug 15 2014 15:11:07 for ESAT by  

 1.8.7
